# Dosimetric comparison of inverse optimization with geometric optimization in combination with graphical optimization for HDR prostate implants

**DOI:** 10.4103/0971-6203.26694

**Published:** 2006

**Authors:** Swamidas V. Jamema, Sherly Saju, Umesh M. Shetty, Siddanna Pallad, D. D. Deshpande, S. K. Shrivastava

**Affiliations:** Department of Medical Physics, Tata Memorial Hospital, Parel, Mumbai - 400 012, India; * Department of Radiation Oncology, Tata Memorial Hospital, Parel, Mumbai - 400 012, India

**Keywords:** Anatomy based inverse optimization, geometric optimization, prostate HDR implant dosimetry

## Abstract

The purpose of this study is to compare geometric optimization (GO) with anatomy based inverse optimization (ABIO). Five patients of carcinoma prostate treated with HDR interstitial brachytherapy had been studied. Post implant CT scans of 5 mm slice thickness were obtained; target volume and other critical structures rectum, bladder and urethra were drawn by the clinician. Plans were obtained with geometric optimization and anatomy based inversed optimization. Anatomy based inverse planning implemented currently in PLATO BPS version 14.2, is based on geometric and dose point optimization and designed to account for the critical structures. Graphical optimization (GrO) is used to fine-tune the distribution ie to reduce the dose to critical structures and to improve the target coverage in both geometric optimization and anatomy based inverse optimization plans. DVH of target, rectum, bladder and urethra were evaluated and compared, dose homogeneity index and conformity index were also evaluated for all the plans. The mean target coverage was 93.9±7%, 90.3±4%, 82±13%, 91.6±3 for different optimization techniques GO, GO_gr, ABIO and ABIO_gr respectively. The target coverage in ABIO is not clinically acceptable. Maximum dose, dose to 2% of the volume of urethra D_2%,U_ was 137±12%, 123.2±2%, 111.5±9, 122.7±4 for GO, GO_gr, ABIO and ABIO_gr respectively. The mean conformity index values were 0.71, 0.76, 0.65, 0.82 for GO, GO_gr, ABIO, ABIO_gr respectively. ABIO_gr has a good conformity over all other optimization techniques. However the difference is not very significant between GO and GO_gr. The mean values of DHI are 0.81, 0.77, 0.65 and 0.75 for GO, GO_gr, ABIO and ABIO_gr respectively. Geometric optimization is highly homogenous compared to all other optimization techniques.

To conclude, target coverage in ABIO is not clinically acceptable. However ABIO followed by graphical optimization is much superior in sparing of critical structures and conformity compared to geometrical optimization. Target coverage is marginally better in GO compared to ABIO_gr. Homogeneity is superior in GO compared to ABIO_gr. However ABIO_gr plans were clinically acceptable with respect to homogeneity. Further, dose escalation to the target is possible with ABIO, without exceeding the tolerance dose to urethra. Clinical correlation of genitourinary toxicity has to be studied.

Treatment planning of prostate cancer is very challenging due to the close proximity of the target to the critical structures such as rectum, bladder and the urethra. Three dimensional conformal radiotherapy (3DCRT) and intensity modulated radiotherapy (IMRT) have reduced the doses to rectum and bladder considerably. However due to the internal organ motion[1] margins have to be given for GTV and CTV to get PTV, which includes part of rectum and bladder, because of which dose to these structures will be higher than the desirable.

The dose to urethra, part of which is inside the PTV is almost impossible to reduce by 3DCRT and even by IMRT. Urethral dose is very significant in controlling the genitourinary toxicities.[2] Brachytherapy definitely has an advantage[3] over 3DCRT and IMRT, because of its steep dose gradient between the critical structures and the target. HDR brachytherapy in particular has an advantage that the dose can be controlled by altering the dwell times of different dwell positions. Dose to the urethra could very well be reduced by interstitial HDR brachytherapy without compromising much on the target coverage. In our center HDR brachytherapy is performed as a boost followed by external beam radiotherapy. Recent studies reported in the literature[4,5] have shown that the results of the combination of tele and brachytherapy to be very good in terms of local control with the disease free survival.

Traditionally geometric optimization (GO) was used for treatment planning of the HDR brachytherapy prostate implants in our centre. HDR temporary implants with GO was considered to be superior over permanent implants[6] for its dose homogeneity and conformity of the dose distribution of the implanted volume. But sparing of critical organs is very crucial in the case of prostate implants and dose to the urethra was still a major challenge while planning with GO. In GO the dwell times of each dwell position could be altered, so we can have many combinations of the dwell time, the challenge is to choose the best possible combination of the dwell times. When anatomy based inverse optimization (ABIO) was implemented in PLATO BPS version 14.2 (Nucletron, BV, Veenendaal Netherlands) HDR prostate implants were planned and found that the dose to urethra was significantly low compared to GO plans. In contrast to GO, ABIO uses target volume and volume of critical organs to be spared with their individual constraints as the input. The limitation of GO of choosing the optimal combination of dwell times from ‘n’ number of combination has been overcome in ABIO. With the constraints of the critical organs as the input the software gives the optimal combination of dwell time as the solution.

It has already been reported[6] that ABIO with graphical optimization to fine tune the distribution has spared the critical organs without compromising the target coverage. Akimoto *et al*.[7] have further reported that toxicities related to urethra had reduced with ABIO when compared to geometric optimization. Lessard and Pouliot[8] had tested simulated annealing inverse optimization algorithm for prostate and gynecological cancers. The ABIO implemented in PLATO BPS version 14.2 is not the algorithm introduced by Lessard and Pouliot which is based on simulated annealing. Graphical optimization (GrO) is another tool in PLATO BPS version 14.2, which helps the planner to shape the isodose distribution by dragging the isodose line using the mouse.

The purpose of this study is to explore the dosimetric superiority of ABIO over GO to reduce the dose to critical structures without compromising the target coverage in the treatment of HDR prostate interstitial implants.

## Materials and Methods

Five patients who were treated with HDR brachytherapy for prostate cancer have been selected for this study. All patients had received 50 Gy/25 fractions of external beam radiotherapy followed by one HDR implant delivering 5 Gy/3 fractions. MUPIT template was used to perform the implants. The average number of implant needles was 18. On the average 70% of the catheters were in the periphery and 30% were around the urethra. During the implant, care was taken not to put the needles in the urthera, needles were kept outside urethra and below the bladder mucosa. Post implant CT scans of 5 mm slice thickness were obtained; target volume and other critical structures rectum, bladder and urethra were drawn by the clinician. Foley's catheter was used to delineate the urethra. Volume of urethra within the target volume and 1cm superior was contoured. Target volume includes prostate without any margin, rectum including the lumen and bladder were contoured. All patients had undergone treatment based on GO on volume. Implant needles were reconstructed using CT reconstruction. The loading pattern of the source depends on the geometry of the target volume. Dwell positions around the target volume + 1 cm margin were activated. Dose points are placed in the target volume based on Paris system rules and normalized on them to get the isodose distribution. GrO was used to reduce the dose to the critical structures by manually clicking the mouse to adjust the isodose lines without compromising the dose homogeneity and coverage of the target volume. Reference isodose line is the one, which covers the target volume adequately. While selecting the reference isodose line the tolerance doses to the critical structures such as rectum, bladder and urethra were respected. The dose to urethra was maintained below 120% of the prescription dose. GO plans give a satisfactory distribution with respect to target coverage, but the limitation of GO is that it is not based on anatomy.

ABIO implemented currently in PLATO BPS version 14.2, is based on geometric and dose point optimization and designed to account for the critical structures. The system requires the constraints of target and other critical structures as the input. The ABIO works in three phases, in the first phase, GO and dose point optimization will be used to obtain the potential of optimization, ie the ability to change the dose distribution while keeping the dose to the prescription points same. The dose points can be divided in to two parts, one set of the points near the periphery of the implant are responsible for the coverage and conformity, whereas the other set of dose points in center will account for the dose homogeneity. In the second phase of ABIO, implant geometry and the critical organs will decide to what extent the optimization is possible, this is quantified and given as the range of maximum dose. In the third phase the planner has to input the value of the maximum dose from the range of the dose and the system will give the optimal plan based on the input value.

When we started planning with ABIO, the dose maximum was set as follows: urethra 120%, rectum and bladder 90%, target 100%, we found that the dose to PTV was very low and at the same time the dose to critical structures were not exceeded the set limits. From the dose volume histogram (DVH), it is found that the percentage volume of the critical structure receiving the dose more than the maximum value set is not clinically significant, which gives us the option of increasing the maximum value as the input. Upon increasing the maximum value of the critical structure for example 180-230% to urethra, the target coverage was considerably improved. From the DVH it was seen that 2-5% of volume of urethra was getting the dose of about 120-150%, where the maximum dose was about 220%, the volume of the dose received by this dose was clinically insignificant. Hence it was decided that the dose received by 2% of volume is considered to be the maximum dose (D_2%,u_). However the actual maximum value given in PLATO would be higher than what we expect it to be. GrO was used wherever necessary to further optimize the plan.

DVH of target, rectum, bladder and urethra were evaluated and compared. Maximum dose to urethra and the dose received by target were noted from each of the plan. In addition to that two indices were computed: conformity index (COIN)[9] describes the normal tissue irradiation, dose homogeneity index (DHI), volume of hotspot with in the treated volume,

$\begin{array}{l} \text{COIN}={\text{c}}_{1}\times {\text{c}}_{2}\\ {\text{c}}_{1}={\text{PTV}}_{\text{ref}}/\text{PTV}\\ {\text{c}}_{2}={\text{PTV}}_{\text{ref}}/{\text{V}}_{\text{ref}} \end{array}$

Where PTV_ref_ refers to the part of the PTV covered by the reference dose and V_ref_ refers to the total volume covered by the reference iso dose. The fraction of the PTV, which is enclosed by the reference isodose, is described by c_1_ and the fraction of the total volume covered by the reference dose that belongs to the PTV by c_2_. COIN can take values between 0 and 1 with 1 associated with full conformity.

The dose homogeneity was analyzed using DHI, the definition is as follows,

$\text{DHI}=({\text{V}}_{100}{\text{-V}}_{150})\text{ D}/{\text{V}}_{100}$

Where V_100_ and V_150_ are the volume of target receiving 100% and 150% of the prescription dose respectively.

## Results and Discussion

Table 1 give the dosimetric parameters of all the optimization techniques. Percentage volume covering the target by the prescription isodose line V_tar_ and the corresponding maximum dose dose to 2% of the volume of urethra (D_2%,U_) urethra were tabulated for GO, GO followed by GrO (GO_gr), ABIO and ABIO followed by GrO (ABIO_gr). The mean target coverage was 93.9±7%, 90.3±4%, 82 ±13%, 91.6±3 for different optimization techniques GO, GO_gr, ABIO and ABIO_gr respectively. D_2%,U_ was 137±12%, 123.2±2%, 111.5±9, 122.7±4 for GO, GO_gr, ABIO and ABIO_gr respectively. From the above results it is seen that the dose to urethra is 111.5% in the case of ABIO, the upper limit constraint for urethra is set 180-250% of prescription dose, yet the dose to urethra has not reached to 120%, hence graphical optimization is carried out to increase to dose to urethra which increases the target coverage.

**Table 1:** Percentage of target coverage by the prescription isodose V_tar_ and dose to 2% of urethra D_2%,U_ tabulated for all the optimization techniques

*Opt parameter*	*GO*		*GO_gr*		*ABIO*		*ABIO_gr*	
				
	*V_tar_*	*D_2%,U_*	*V_tar_*	*D_2%,U_*	*V_tar_*	*D_2%,U_*	*V_tar_*	*D_2%,U_*
1	95.5	142.5	89	130.3	90	119.9	92.9	121.1
2	96.3	137.1	95.	129.1	81.3	111.1	94.2	119.0
3	87.2	125.7	86.7	131.7	69.5	103.6	89.1	123.2
4	94.5	133.4	92.9	133	94	120.4	91.5	125.1
5	96.0	149	87.7	131.7	74.7	102.6	90.4	125.1
mean	93.9±7%	137±12%	90.3±4%	131.2±2%	82±13%	111.5±9	91.63	122.7±4

Geometric optimization GO, geometric optimization with graphical optimization GO_gr, anatomy based inverse optimization ABIO and anatomy based inverse optimization with graphical optimization ABIO_gr

**Figure 1:**
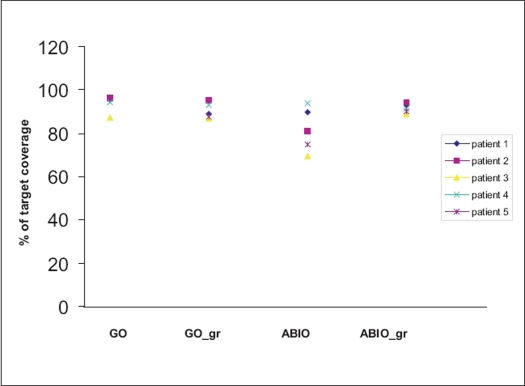
Target coverage for different patients as a function of different optimization techniques. Geometric optimization (GO), geometric optimization with graphical optimization (GO_gr), anatomy based inverse optimization (ABIO) and anatomy based inverse optimization with graphical optimization (ABIO_gr)

**Figure 2:**
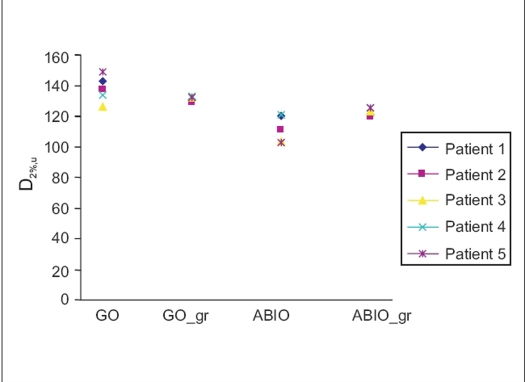
Dose to 2% of urethera (D_2%,U_) for different patients as a function of different optimization techniques: geometric optimization (GO), geometric optimization with graphical optimization (GO_gr), anatomy based inverse optimization (ABIO), anatomy based inverse optimization with graphical optimization (ABIO_gr)

**Figure 3:**
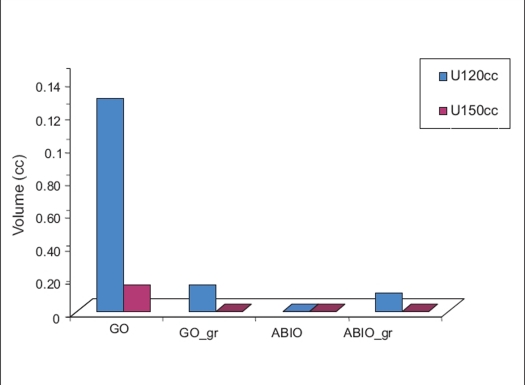
Volume of urethra (cc) receiving 120 and 150% of the prescription isodose for different optimization techniques: geometric optimizations (GO), geometric optimization with graphical optimization (GO_gr), anatomy based inverse optimization (ABIO) and anatomy based inverse optimization (ABIO_gr)

**Figure 4:**
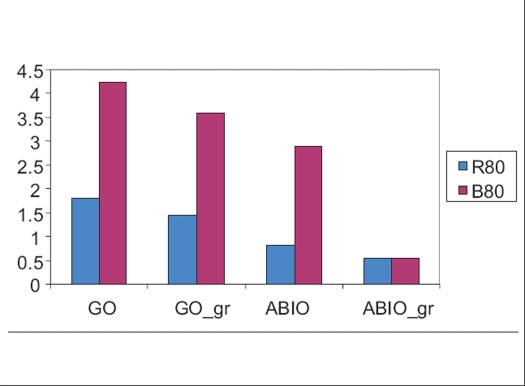
Volume (cc) of rectum and bladder receiving 80% of the prescription dose for different optimization techniques: geometric optimizations (GO), geometric optimization with graphical optimization (GO_gr), anatomy based inverse optimization (ABIO) and anatomy based inverse optimization (ABIO_gr)

Figure 1 gives the target coverage for different optimization techniques. The difference between GO and GO_gr is not very significant for target coverage, 93.9±7% and 90.3±4% for GO and GO_gr respectively, but decrease of target coverage was noticed for ABIO 82%±13 compared to 91.6%±3 for ABIO_gr. Figure 2, gives the dose received by urethra by different optimization techniques. Figure 3 gives the volume of urethra received by 120 and 150% of prescription dose. GO plan had produced the highest dose to the urethra, 137%±12. However using the graphical optimization tool, dose to the urethra was reduced to 131.2%±2. While doing graphical optimization to reduce the dose to urethra, target coverage also was reduced. To reduce the dose to urethra by a mean of 14%, the target coverage has to be compromised by mean 4%. Optimal reduction of dose to urethra without compromising the target coverage depends on the clinician's decision and the planner's experience. In the case of ABIO, the dose to urethra has not received its maximum tolerance value in spite of giving a high dose constraint. Considering the dose to urethra, in ABIO there is still a room for increasing the urethral dose, hence graphical optimization was used to improve the target coverage while keeping the maximum dose to urethra below 120% of the prescription isodose. Figure 4 gives the dose to rectum and bladder for different optimization techniques, the mean volume of rectum and bladder receiving 80% of the prescription dose is compared. In ABIO_gr the dose to rectum and bladder is least compared to all other optimization techniques.

**Figure 5:**
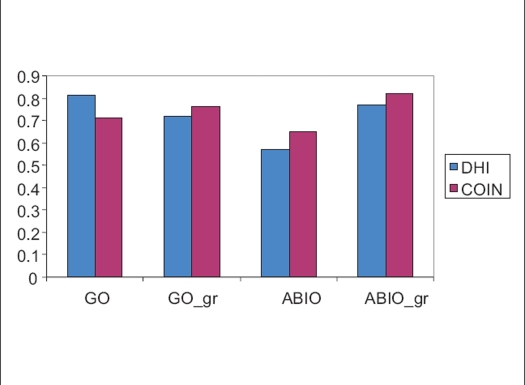
Dose homogeneity index and conformity index for different optimization techniques: Geometric optimization (GO), geometric optimization with graphical optimization (GO_gr), anatomy based inverse optimization (ABIO) and anatomy based inverse optimization (ABIO_gr)

To evaluate the normal tissue irradiation, Conformity index was compared for all optimization techniques [Figure 5], the mean conformity index values were 0.71, 0.76, 0.65, 0.82 for GO, GO_gr, ABIO, ABIO_gr respectively. ABIO_gr has a good conformity over all other optimization techniques. However the difference is not very significant between GO and GO_gr.

Figure 5 gives the homogeneity index and conformity index for different optimization techniques. The mean values of DHI are 0.81, 0.77, 0.65 and 0.75 for GO, GO_gr, ABIO and ABIO_gr respectively. Geometric optimization is highly homogenous compared to all other optimization techniques. However GO_gr and ABIO_gr are also homogenous, when we were in the learning curve of GrO technique, we used to get highly inhomogeneous distribution, but the planner has to know when to stop dragging the isodose line so that the homogeneity of the implant is maintained.

GrO is a powerful tool that allows the user to drag the isodose line wherever required, while doing so in one cross section of the image, the isodose distribution will change in other images and alter the distribution which will change the homogeneity, conformity and even the dose to other critical structures. Hence a careful analysis of the distribution has to be carried out, before altering the distribution. The hot spots can be accepted at some part of the implant, whereas near the urethra the dose was kept below 120% of the prescription isodose line. In most of the cases instead of dragging the isodose line, the dwell times were changed manually depending on the dose distribution.

In this study our aim was to evaluate ABIO implemented currently in PLATO BPS with conventional GO with GrO as a tool to fine-tune the distribution to reduce the dose to urethra. From the above results it can be concluded that ABIO has reduced the dose to urethra significantly when compared to GO. The mean dose to urethra with just GO is 137±12% and target coverage is 93.9±7% of the prescription dose, the highest among the four optimization techniques. Geometric optimization method produces a highly homogenous distribution, which means that both target and critical structures receive the same dose, however conformity is inferior which represents that normal tissue irradiation is high in GO. When GrO was used on GO plans, the dose to urethra was reduced by 6%, thereby reducing the target coverage also by 2%.

To begin with, ABIO plans were very inferior compared to GO, in terms of target coverage but superior to the dose received by the urethra. The dose to urethra was very much less than what we had expected. When the maximum dose constraint was set at 120%, dose calculated by the system was nearly 100%, which gives us the room to increase the constraint for urethra so that target coverage also could be improved. When the dose constraint of urethra had increased to about 230% or so, the target coverage and the dose to 2% of urethra had been within the expected limits. Further the various dose levels 30%, 50%, 80%, 90% received by the volume of the urethra is much less compared to GO and GO_gr. Graphical optimization is a powerful tool to fine tune the distribution in both GO and ABIO. From the above conclusion it is learnt that ABIO may produce better plans with dose volume constraint, than just maximum dose constraint to the critical structures. The optimization by ABIO presently implemented in PLATO BPS is not clinically acceptable in terms of target coverage, however with due changes with modified constraints and with graphical optimization tool clinically acceptable plans could be produced. The method ABIO used here is not the inverse optimization algorithms described in the literature,[8] which is based on multi objective optimization algorithm and simulated annealing. Comparison studies[5,7] had shown that these optimization algorithms provide better plans with respect to target coverage and dose to critical structures. Our study also shows that the dose to urethra could be reduced without compromising much on target coverage same as that of above studies which uses simulated annealing and multi objective optimization algorithms. But the graphical optimization tool takes long time and need an experienced planner to produce a plan that is clinically acceptable, otherwise the homogeneity and the conformity of the implant will suffer to a larger extent, much to the contrary, that the inverse planning by simulated annealing hardly takes few minutes and the plan is not subjected to the experience of the planner. Table 2 gives the summary of all the four optimization techniques, ABIO_gr is acceptable with reference to all the parameters, however the homogeneity may not be acceptable in some situations, but with experience it should be possible to obtain clinically acceptable homogenous plans.

**Table 2:** The summary of the findings is tabulated: target coverage, dose homogeneity index, conformity index, volume of urethra receiving 120 and 150% of prescription isodose and volume of rectum and bladder receiving 80% of prescription dose is compared for all the different types of optimization techniques

	*GO*	*GO_gr*	*ABIO*	*ABIO_gr*
Target coverage	(93.9±7%) √√	(90.3±4%) √	(82±13%) ××	(91.6±3) √
DHI	(0.81)√√	(0.77) √	(0.65) ×	(0.75) √
COIN	(0.71) ×	(0.76) √	(0.65) ×	(0.82) √√
U_120_ cc	(0.12) ×	(0.015) √	(0) √	(0.01) √
U_150_ cc	(0.015) ×	(0) √	(0) √	(0) √
R_80_ cc	(1.82) ×	(1.44) √	(0.8) √	(0.54) √
B_80_ cc	(4.24) ×	(3.59) √	(2.89) √	(0.537) √

Geometric optimization GO, geometric optimization with graphical optimization GO_gr, anatomy based inverse optimization ABIO and anatomy based inverse optimization with graphical optimization ABIO_gr.√ symbol represents that is clinically acceptable × symbol represents that is not clinically acceptable

## Conclusion

Target coverage in ABIO is not clinically acceptable. However ABIO_gr is much superior in sparing of critical structures and conformity compared to GO. Target coverage is marginally better in GO compared to ABIO_gr. Homogeneity is superior in GO compared to ABIO_gr. However ABIO_gr plans were clinically acceptable with respect to homogeneity. Further dose escalation to the target is possible with ABIO_gr, without exceeding the tolerance dose to urethra. Clinical correlation of genitourinary toxicity has to be studied.
